# Bladder tissue characterization using probe‐based Raman spectroscopy: Evaluation of tissue heterogeneity and influence on the model prediction

**DOI:** 10.1002/jbio.201960025

**Published:** 2019-12-02

**Authors:** Eliana Cordero, Jan Rüger, Dominik Marti, Abdullah S. Mondol, Thomas Hasselager, Karin Mogensen, Gregers G. Hermann, Jürgen Popp, Iwan W. Schie

**Affiliations:** ^1^ Department of Spectroscopy and Imaging Leibniz Institute of Photonic Technology (Leibniz‐IPHT) Jena Germany; ^2^ Department of Health Technology Technical University of Denmark (DTU) Roskilde Denmark; ^3^ Department of Urology Herlev Hospital Herlev Denmark; ^4^ Institute of Physical Chemistry, Friedrich Schiller University Jena Jena Germany; ^5^ Department of Medical Engineering and Biotechnology University of Applied Sciences, Jena Jena Germany

**Keywords:** bladder cancer, imaging‐based Raman, PLS‐LDA, Raman probe, Raman spectroscopy

## Abstract

Existing approaches for early‐stage bladder tumor diagnosis largely depend on invasive and time‐consuming procedures, resulting in hospitalization, bleeding, bladder perforation, infection and other health risks for the patient. The reduction of current risk factors, while maintaining or even improving the diagnostic precision, is an underlying factor in clinical instrumentation research. For example, for clinic surveillance of patients with a history of noninvasive bladder tumors real‐time tumor diagnosis can enable immediate laser‐based removal of tumors using flexible cystoscopes in the outpatient clinic. Therefore, novel diagnostic modalities are required that can provide real‐time in vivo tumor diagnosis. Raman spectroscopy provides biochemical information of tissue samples ex vivo and in vivo and without the need for complicated sample preparation and staining procedures. For the past decade there has been a rise in applications to diagnose and characterize early cancer in different organs, such as in head and neck, colon and stomach, but also different pathologies, for example, inflammation and atherosclerotic plaques. Bladder pathology has also been studied but only with little attention to aspects that can influence the diagnosis, such as tissue heterogeneity, data preprocessing and model development. The present study presents a clinical investigative study on bladder biopsies to characterize the tumor grading ex vivo, using a compact fiber probe‐based imaging Raman system, as a crucial step towards in vivo Raman endoscopy. Furthermore, this study presents an evaluation of the tissue heterogeneity of highly fluorescent bladder tissues, and the multivariate statistical analysis for discrimination between nontumor tissue, and low‐ and high‐grade tumor.
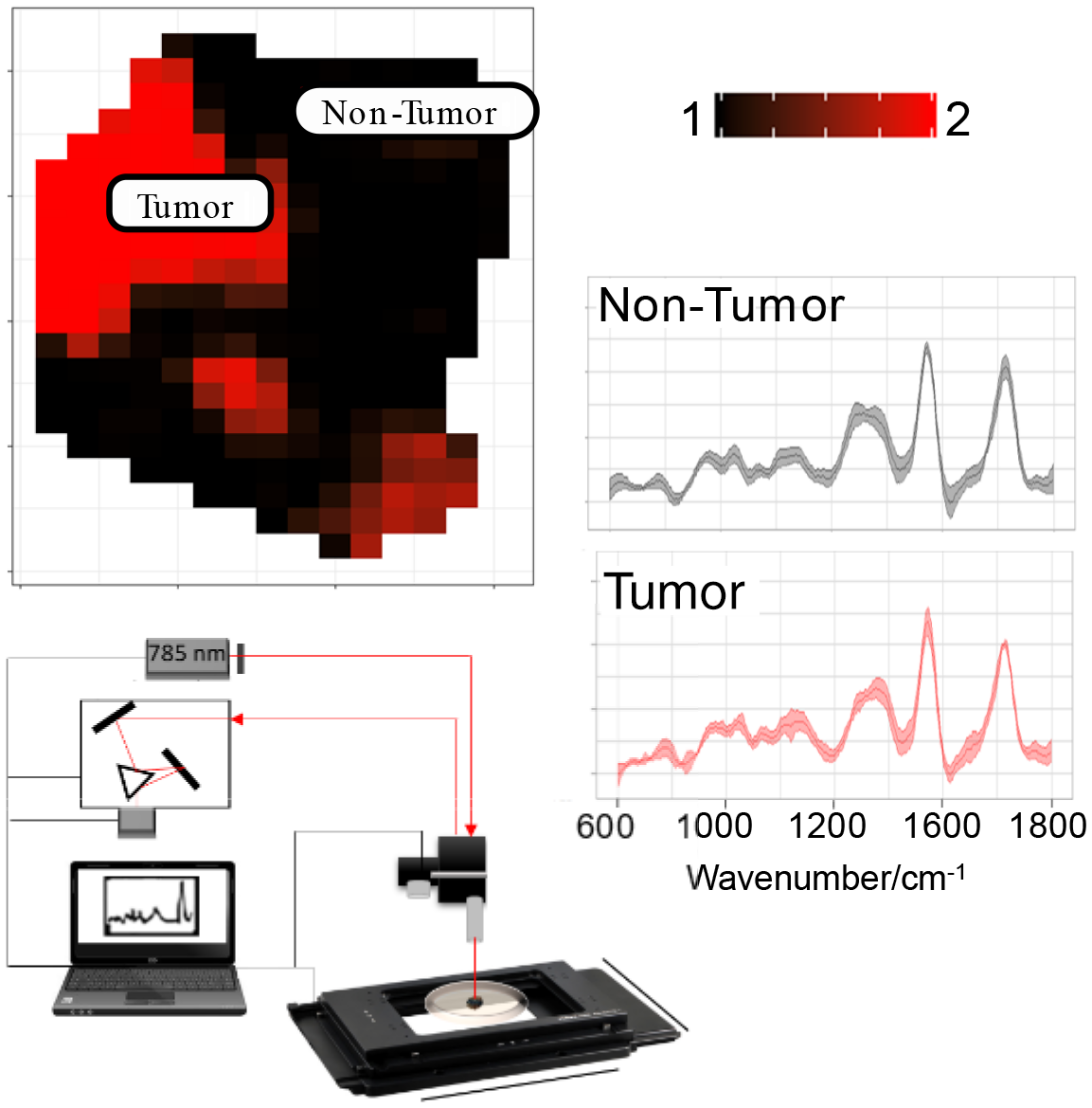

## Graphical abstract

The newly developed Raman‐platform for label‐free characterization of ex vivo bladder tissue enables a comprehensive characterization biopsy samples, that is, differentiation of tumor and nontumor regions, and also the grading of the tumor.

## 1 INTRODUCTION

Bladder cancer is ranking as the ninth most frequently diagnosed epithelial cancer worldwide. In 2012, around 549 393 new bladder cancers cases were reported globally, out of which 200 000 cases were fatal, and approximately 75% of the deceased were males 1, 2. About 75% of the patients suffer from nonmuscle invasive bladder cancer displaying favorable prognosis, although 30%‐80% of cases will recur 3. Bladder cancer histopathological diagnosis is based on stage and grade of the tumor, where the stage refers to tumor invasion into the bladder wall and grade to aggressiveness of the cells. Stage and grade are judged by pathologist's examination of an excisional tissue biopsy obtained via an endoscope inserted through the urethra to the bladder, having the patient most often in general anesthesia in the operating theater. The tissue from suspected abnormal areas of the bladder is sliced and stained for further morphological evaluation by a pathologist. To monitor progression and recurrence a frequent screening is required, making the current bladder cancer diagnosis and treatment one of the priciest medical practices with average lifetime costs estimated at over $230 000 per patient 4, 5, 6. The histopathological procedure provides morphological tissue features at the intra‐ and intercellular level, nevertheless, the underlying biochemical information is not assessed 7. Moreover, the diagnosis is not available instantly due to histological/cytological preparations preceding final microscopy by the pathologist. In order to improve treatment, real‐time differentiation between healthy and tumor tissue, high‐ and low‐grade lesions at early stage is urgently needed 8, 9, 10. In the past two decades new optical methods for clinical diagnostics, such as fluorescence endoscopy 11, optical coherence tomography 12, narrow band imaging 8, and others, have emerged. Most of these techniques effectively provide contrast to detect tumor lesions and allow for differential diagnostics, that is, tumor vs healthy tissue, but largely lack the means to assess the biomolecular, which may assist the urologist to decide treatment without delay. Knowing the molecular fingerprint of cells not only allows for precise diagnostic characterization of the tumor, but also enables new pathological insight into the disease progression. Raman spectroscopy has emerged as an incipient tool for in vivo diagnostics, which provides a comprehensive and label‐free biochemical characterization of tissue samples 13. The method has been widely used in clinical ex vivo and in vivo investigations for the diagnosis of inflammatory diseases and cancers in different organs 13, 14, 15, 16, 17, 18, 19, 20, 21, 22, 23, 24, 25, 26, 27, 28, demonstrating the great potential for label‐free histopathology 29, cytology 30, biopsy surgical targeting and monitoring studies. This spectroscopic technique has readily been used to characterize bladder tissue by De Jong et al and Stone et al in 2002 13, 31, demonstrating its capability to distinguish tumor malignances in epithelial tissues 19. A review on the applications of Raman spectroscopy for the interrogation of bladder tissue for cancer diagnosis is summarized in 32.

Raman spectroscopy has been applied in vivo to characterize tumor tissue, assisting surgeons during transurethral resection of tissue to differentiate on site malignant tumor 33. As a first instance of in vivo bladder characterization, Draga et al reported the ex vivo and in vivo characterization of bladder tissue, employing a fiber‐optic Raman probe to detect tumor bladder from normal bladder with a sensitivity of 85% 34. Notwithstanding, tumor resection of biopsies is still invasive; the target is to adapt Raman spectroscopy to an endoscope in order to minimize the invasion during the tissue inspection in surgery. The present study explicitly aims at providing an in‐depth characterization of bladder cancer and outlines strategies for data processing and establishes parameters for future in vivo label‐free diagnosis 35. Therefore, greater extend of ex vivo investigations are crucial to further move this technology into this direction, in this connection the presented study plays an essential role.

The translation of Raman spectroscopy as a clinical standard tool to assist current diagnosis of bladder tumor grading still must tackle technological and methodical challenges. For instance, proper correction of tissue autofluorescence issue, routines to validate the robustness of the system for clinical use of the equipment, and the consensus on optimal data preprocessing methods will have to be further investigated 36.

The aim of the present study is to demonstrate the feasibility of Raman spectroscopy to further complement clinical trials by differentiating the tumor grading of bladder biopsies and correlating the main changes in molecular constituents to characterize the tissue heterogeneity. We provide a comprehensive overview of sample and data handling, followed by detailed description of the implemented system. We elucidated problems of tissue heterogeneity, which can significantly reduce the performance of a model. In addition, we present a comparison between model‐based prediction for tumor and non‐tumor and changes in the molecular signatures associated with the pathological differences. Our presented evaluation will help to improve the comprehension of the molecular differences between underlying molecular changes in bladder pathology.

## 2 MATERIALS AND METHODS

### 2.1 Study population and procedures

Biopsies were obtained from patients, admitted to the urology department in Herlev hospital, suffering from bladder tumor or suspicion of bladder tumor disproved during surgery. The clinical study protocol was approved by the local Danish ethical committee No: H‐17015549. Flow of sensitive data was secured and approved by the Danish Data Protection Agency via data management agreements between research centers. After patients were informed and written consent was conferred, biopsies were obtained at the operating theater during transurethral resection of bladder tumors. The biopsies were obtained from healthy bladder wall and from bladder tumors. Each biopsy was divided into two parts, of which one biopsy was sent for a histopathological diagnosis and the other was placed on acetate paper and saline buffer added for spectroscopic examination. In contrast to previously reported studies 13, 37, an entire extracted biopsy without thin‐sectioning was used in this study with the purpose of maintaining the integrity of the tissue, since sectioning and staining can severely alter the structure of the biopsy. The summary of the present pathologies is outlined in Table S1.

**Figure 1:**
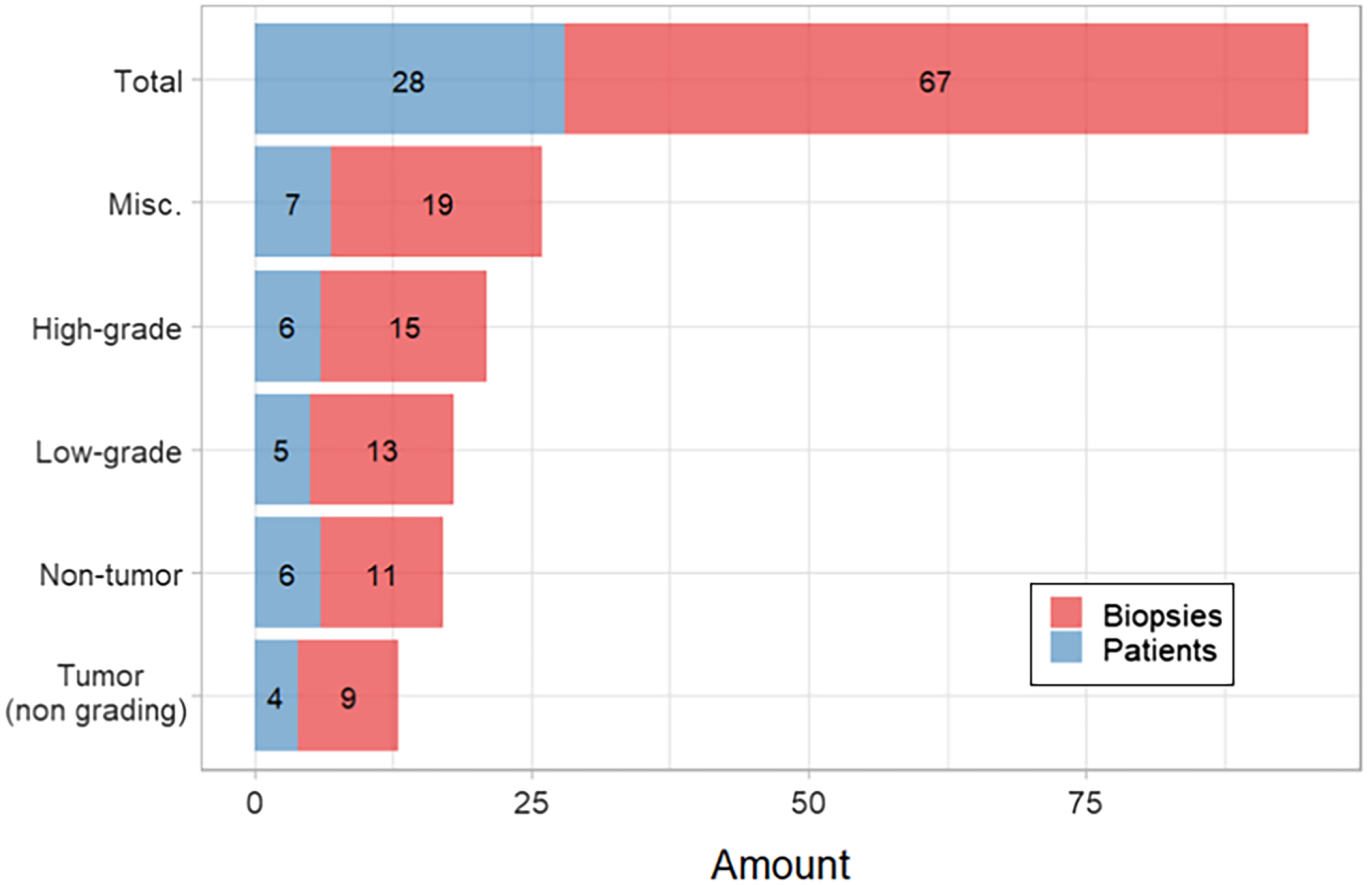
Summary and breakdown of patients and biopsies. Misc. refers to miscellaneous nonbladder tumor histopathology (four benign prostatic tissues, two cancer prostate tissue, six biopsies with unknown histopathology), and biopsies measured under different experimental conditions. Biopsies belonging to this group were not selected to train the model. Tumor (nongrading) refers to histopathological assignment indicating tumor without grading. This data was used just to create the models that differentiate tumor and nontumor regions

A total of 67 biopsies were obtained from 28 patients with bladder tumor (8 females; 20 males; median age: 73 years) from which 19 biopsies from seven patients were excluded from the data analysis due to either being of other tissue, that is, benign prostate (n = 4), prostate cancer (n = 2), unknown histopathology (n = 6) or measured under different experimental conditions (n = 7) (Figure 1). Of the remaining biopsies a total of 42 biopsies were examined immediately after surgery and six were frozen at −80°C, and analyzed at a later time point.

### 2.2 Setup description

**Figure 2:**
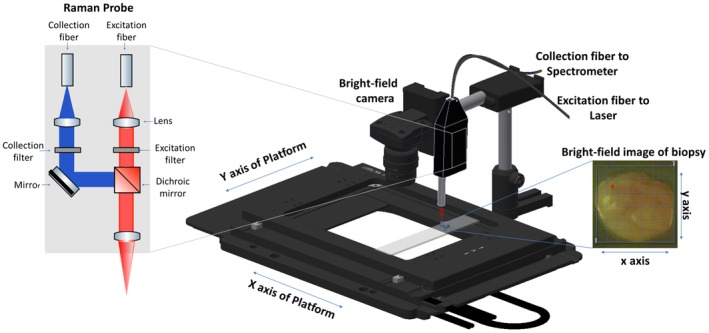
Schematic representation of the Raman imaging system combined with a Raman fiber‐optic probe. The optical design of the fiber‐optic Raman probe is indicated in more detail on the left‐hand side

Raman spectra were collected on a custom‐made Raman system equipped with a fiber‐optic Raman probe (InPhotonics, RPB), which was connected to a 785‐nm single‐mode excitation laser (XTRA, Toptica) with a nominal output power of 300 mW, and a spectrometer (IsoPlane 160, Princeton Instruments) equipped with a 400 groves/mm grating and a back‐illuminated deep depletion CCD camera (PIXIS400, Princeton Instruments) with a 1340 × 400 imaging array and 20 μm × 20 μm sized pixels. The excitation light was filtered inside the probe to remove unwanted background contributions from the delivery fiber and focused by a lens into a spot of 100 μm. The generated Raman signal was collected by the same lens. The signal was separated from the excitation with a dichroic mirror, and then focused into the entrance aperture of a multimode collection fiber with a core diameter of 200 μm and an numerical apperture (NA) of 0.22, which is connected to the spectrometer. The samples were placed on a calcium fluoride (CaF_2_) slide (Crystal, Germany) that was mounted on the motorized translational stage (MLS203, Thorlabs). The setup is illustrated in Figure 2.

Raman images of the biopsies were acquired by raster‐scanning the sample, while keeping the probe stationary. A conventional CMOS camera (DCC1545M, Thorlabs), which was located next to the Raman probe, allowed the acquisition of a brightfield image of the sample and the selection of a region of interest (ROI) for the Raman measurements. The acquisition of Raman spectra from the biopsy was performed automatically, using in‐house software for instrument controlling written in LabView. Each of the samples was placed on the CaF_2_ slide with the urothelial surface pointing to the Raman probe. Similar to previously reported studies, a 785‐nm excitation wavelength was selected to avoid high fluorescence 38, 39, with an excitation power of 100 mW, which allows obtaining a sufficient signal to noise ratio, without any obvious damage to the tissue. Each spectrum was acquired at an acquisition time of 3 seconds, but the total spectral collection time was dependent on the ROI size. For the averaged sample size of approximately 4 mm^2^ the measurement of 400 spectra took around 20 minutes.

### 2.3 Data analysis

All data pretreatment and analysis steps were performed using the RStudio software for statistical computing and graphics 40, 41. The data import, export, the preprocessing algorithms and the development of the classification model were performed using hyperSpec and cbmodels packages 42.

#### 2.3.1 Preprocessing

**Figure 3:**
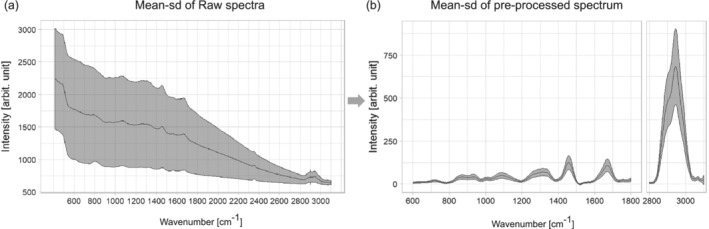
Mean and SD spectra of (A) raw Raman spectra for the entire dataset and (B) a mean Raman spectrum after the preprocessing

Raman spectra of bladder tissue exhibit very high tissue autofluorescence (Figure 3A). To extract the Raman signal from the raw spectra the data was preprocessed, that is, calibrated and corrected for cosmic spikes and background contributions, respectively. The wavenumber calibration was performed using the relative peak positions of *N*‐acetyl‐*p*‐aminophenol powder (Acetaminophen, Sigma‐Aldrich) and intensity calibration was performed by using the reference spectrum of a white light source standardized by the National Institute of Standards and Technology (Kaiser HCA calibration accessory). The correction for the constant offset bias and the dark current was implemented by subtracting a recorded dark spectrum. Following this, cosmic spikes were removed using a correction algorithm developed by Ryabchykov et al 43. The calibrated spectra were noise‐filtered using the prcomp function of the stats package in R, followed by a Savitsky‐Golay filtering 44.

As introduced in this section, one of the main challenges for the data preprocessing is the high fluorescence background, as it can be observed in Figure 3A. This background mainly originates from the presence of endogenous fluorophores, such as pyridinic (NADPH) and flavin coenzymes as well as collagen and elastin from the extracellular matrix 45. Besides the high autofluorescence from the tissue that obscures the Raman signal, there is some additional background from the fiber probe. Among the background correction methods asymmetric least squares (ALS) 46, the modified polynomial fitting of Lieber and Mahadevan‐Jansen 47, statistics‐sensitive nonlinear iterative peak‐clipping algorithm (SNIP) correction 48 and extended multiplicative signal correction (EMSC) were tested. Figure S1 displays the individual results for a visual comparison. The best performance for background correction was achieved by EMSC, Figure S1A, and is based on a least squares fitting of predefined background spectra, *n*‐order polynomials, and pure components spectra 49. To provide a comparison, the other methods are also shown: ALS corrected spectra, Figure S1B, presented overfitting in the low‐ and high‐wavenumber region and background contributions from fiber and substrate were not removed, the same is observed applying the polynomial fitting approach (Figure S1C). The SNIP correction, Figure S1D, can remove background coming from fiber and substrate as EMSC does, however, it is slower than EMSC and the SD due to the background is higher in comparison to EMSC. Following the background correction, the data was normalized to unity. The resulting mean spectrum with the spectral information from 600 to 3100 cm^−1^ is shown in Figure 3B, where the main spectral contributions are related to proteins, collagen, nucleic acids and lipids.

#### 2.3.2 Classification model

The classification was performed combining partial least squares regression (PLS) as dimension reduction technique with linear discriminant analysis (LDA) to differentiate three main classes from the biopsies: nontumor (NT) tissue, low‐grade (LG) and high‐grade (HG) tumor. The combination of the PLS and LDA classifier allows to have an insight in the underlying interclass differences in the molecular signature via loadings and weights. In addition, the combination of the methods has demonstrated that it can cope with large variable to sample ratios 50.

In the PLS‐LDA model, the linear discriminant (LD) classifier uses the number of components determined by the partial least squares, also known as the latent variables, as input space. The dimension reduction technique helps to select relevant variables correlating the best with the class attributes 50, 51. The partial least squares regression was performed using the function plsr of the pls package 52. The predictor matrix was based on several mean spectra per biopsy and the response vector contained the histopathological assignment. The number of components was determined based on leave‐one‐out‐cross‐validation, while assessing the highest correlation vs the least error 53.

#### 2.3.3 Cross‐validation

**Figure 4:**
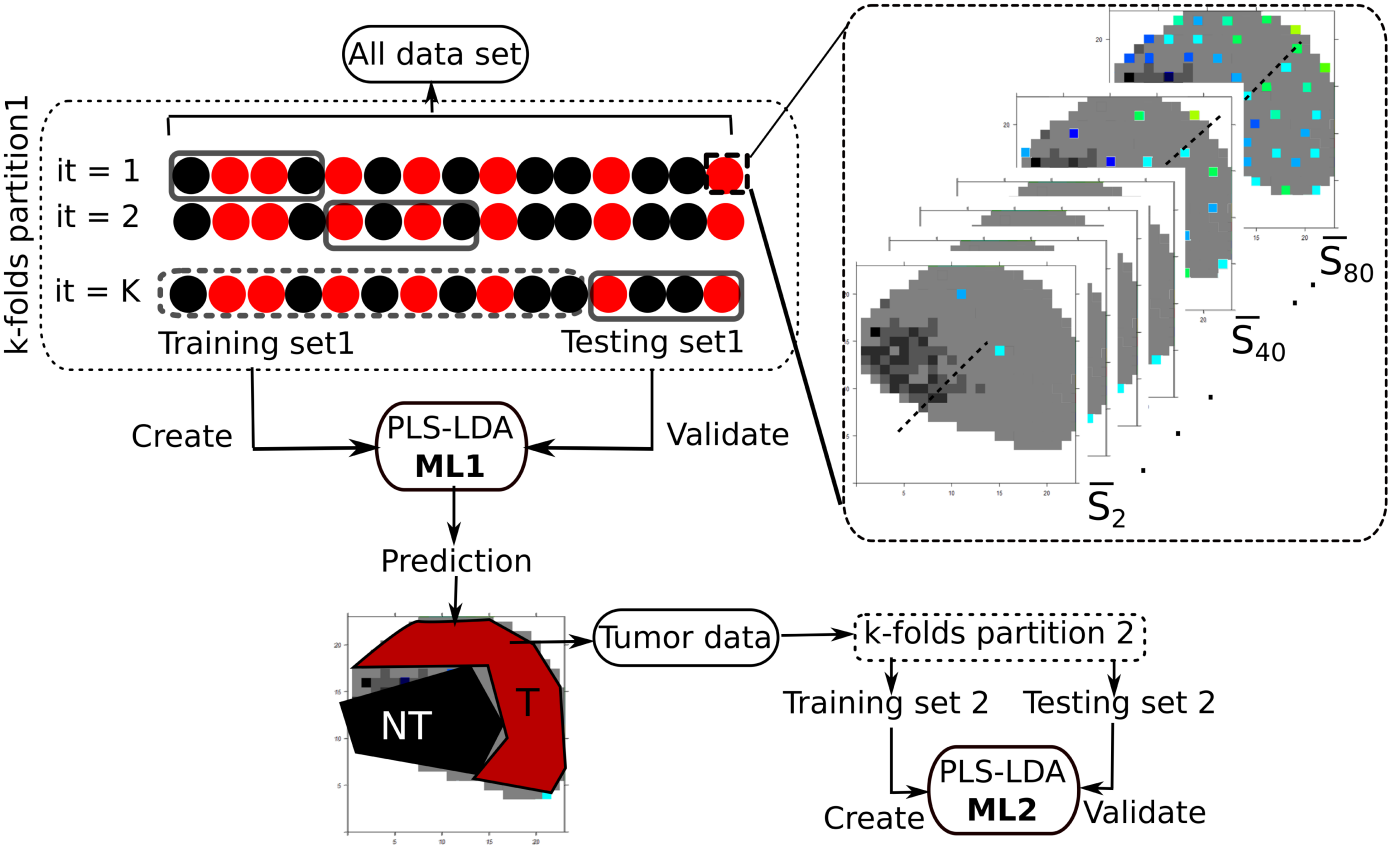
Workflow of the data splitting method used for the *k*‐fold cross‐validation of the models. Hierarchical splitting of data, the data was split in fivefold groups to create (training set 1) and validate (testing set 1) the first level models (ML1): nontumor and tumor; the models of the first level are used to select the tumor areas of the tumor data, just the tumor data resulted from the predictions of ML1 is further partitioned in fivefold to obtain 5 groups of training set 2 and testing set 2, creating and validating the second level models (ML2), respectively. The zoomed area represent how the mean set for each biopsy is obtained, random points from 1 to 80 are selected and for each of the random groups a mean spectra is determined and each biopsy has a group of 80 mean spectra

To prevent overfitting of the classifier, cross‐validation (CV) was performed by applying a hierarchical scheme for classification models, as presented in more detail by Guo et al 54. Hereby, a two‐layer CV was applied where the first layer or internal CV, known as training dataset, was employed to construct the model and the external CV or testing dataset was used for validation. The performance of the classification model was validated by applying hierarchical splitting of the data, where a two‐level model was created, and is referred in the rest of the text as model level 1 (ML1) for tumor and NT differentiation, while model level 2 (ML2) refers to the differentiation of LG and HG tumor, respectively. To test the influence of the sampling area for ML1, between 1 and 80 spectra were taken from random pixel locations of each biopsy, and a mean spectrum was calculated. The dataset was partitioned into fivefold with 10 iterations, resulting in 50 different models for each set, as illustrated in Figure 4. The prediction for the tumor area is displayed in Figure S3, where the prediction for the tumor and NT location is indicated for a typical biopsy, and an indicated number of spectra. To better summarize the results, the prediction of the models for the indicated number of spectra is plotted as the ratio of tumor region and total region to the number of spectra used to build the model (Figure S4). In most situations the ML1 model performs independently of the number of spectra used. To ensure that only spectra from tumor areas enter the subsequent modeling ML2 phase, the predictions for each single spectrum per biopsy were aggregated and the respective spectrum was considered as “tumor” at a mean prediction value above 1.5 and as “NT” at a value below 1.5, respectively. Spectra with the predictive value 1.5 were not assigned to any group. This range was selected by calculating the kernel density of the mean prediction of each biopsy, where it was found that for this range the likelihood for belonging to any of the group NT (1) or tumor (2) is 0. Shortly, the calculated mean prediction was performed on each spectrum of each biopsy and just the set of spectra predicted as tumor was subsequently used for next modeling phase, this is represented in Figure 4. Based on selected spectra from malignant tissue the ML2 were created by using fivefold CV with 10 iterations. The whole workflow is described in more detail in Figure 4.

## 3 RESULTS AND DISCUSSION

**Figure 5:**
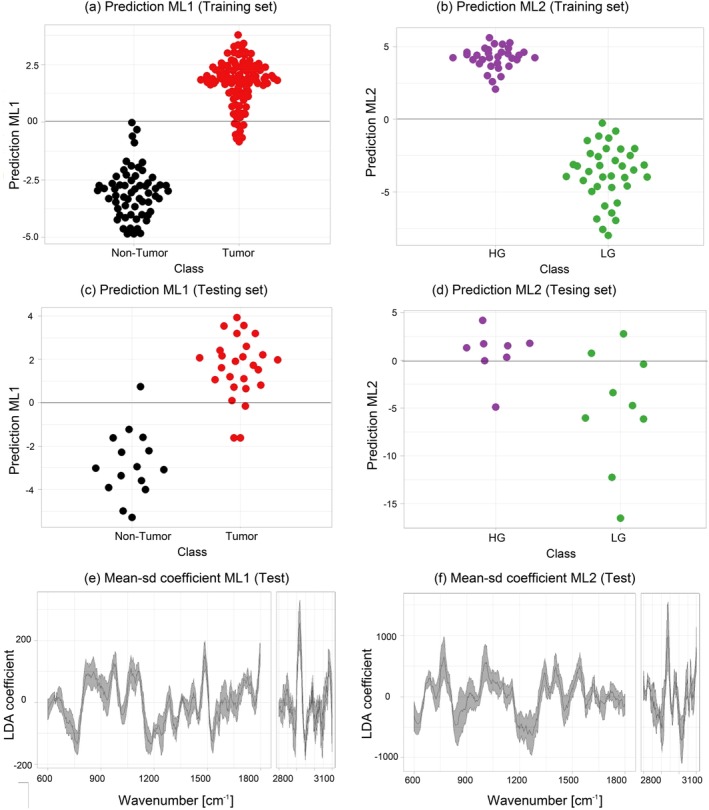
Beeswarm plots of the LDA scores of classification models mean ± SD of the coefficients for a single iteration. Figure 5A,B beeswarm plot after predicting the models level 1 with the training sets 1 and models level 2 with the training sets 2, respectively. Figure 5C,D beeswarm plot after validating the models level 1 with the testing sets 1 and the models level 2 with the testing sets 2, respectively. Figure 5E,F mean ± SD of the coefficients for the predictions after validating ML1 and ML2

**Figure 6:**
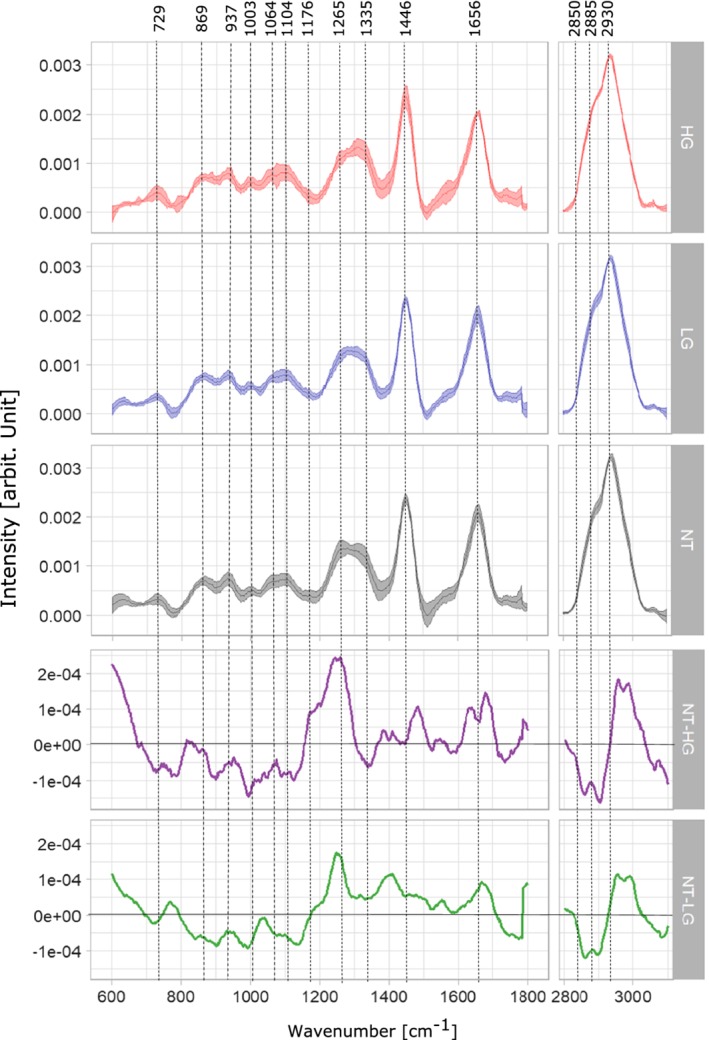
Mean Raman spectra with corresponding standard deviation of biopsies diagnosed as nontumor (NT, black), low‐grade (LG, blue) and high‐grade (HG, red) tumor tissue, respectively. The two bottom panels are difference spectra derived from mean spectra from NT and HG tumor and the difference spectrum between the mean of NT and LG tumor. The vertical lines indicate major bands

In total 67 biopsies were measured out of which 48 were used to create and validate the ML1 model and of those 28 biopsies were used to generate and validate ML2. Spectra from biopsies were assigned to three groups according to the histopathological grading of the tissue, that is, NT, LG and HG tumor, Figure 5. The mean spectra and SD of each group of spectra have been calculated and plotted in Figure 6. The main bands associated to lipid, collagen, protein and nucleic acids have been labeled to allow an assignment of the main differences between each group and its molecular constituents. The goal of the investigation was, firstly, to characterize and to discriminate bladder cancerous tissue and to perform tumor grading, using an in‐house developed fiber probe‐based Raman‐imaging platform, which effectively mimics the in vivo conditions. Secondly, to provide and compare a comprehensive data preprocessing and analysis workflow that can deal with commonly occurring background contributions, for example, fiber and autofluorescence background, and to introduce a robust analysis strategy, which extracts compressive information from the biopsies and classifies tumor and cancerous grade in bladder. Thirdly, to evaluate the effects of intrinsic tissue heterogeneity on the performance of the models and to explore the spatial variation of Raman bands representative for dominant constituents of cancerous/healthy bladder tissue.

### 3.1 Chemometric modeling of biopsy grading

As outlined in Section 2.3.3, a two‐level PLS‐LDA classification model was built to differentiate between tumor from NT ML1 and LG from HG tumor tissue ML2. Figure 5A,C shows the beeswarm plots of the LD scores for the training and testing dataset of a fivefold iteration for ML1, respectively. The figures indicate that the model performs very well to separate tumor (red) from NT (black) biopsies with a sensitivity of 92% and a specificity of 93%, Table 1. The achieved values to discriminate tumor and NT tissue are well within the range reported in previous studies 31, 32. Despite that grade differentiation is not as high, similar results were also reported 32, 55. While most of the reported studies used microscopy setups, here measurements were performed using a small hand‐held probe, which allowed to significantly reduce the footprint of the entire device. The achieved classification values were also potentially influenced by the heterogeneity of the measured biopsies, and the fact that, even though the biopsies were taken in close vicinity slight differences which could have influence the validation of the model, might have been present.

**Table 1:** Statistics of the model by class

Class	ML1 (%)	ML2 (%)
Sensitivity	92	85
Specificity	93	83
Accuracy	92	84
Confidence interval	(89‐95)	(78‐89)

*Note*: The confidence interval is calculated for the accuracy.

The mean and the SD of respective LD coefficients of ML1 are shown in Figure 5E. It is important to point out that for ML1 the negative LD coefficients correlate with the bands that indicate features related to NT tissue, while the positive coefficients values indicate bands related to tumor tissue. Positive features around 1299 and 1313 cm^−1^ indicate dominant presence of lipid bands in tumor tissue, and negative features in the same region indicate the dominant presence of collagen in NT tissue. The performance of the ML2 is represented in Figure 5B,D where the LDA scores are shown in a beeswarm plot and a clear separation between the LG (green) and HG (lila) classes is observed. The negative coefficients in Figure 5F are indicators of spectral features to distinguish LG from HG tumor. ML2 achieve an adequate tumor grading in terms of predictive performance with a sensitivity of 85% and an overall model accuracy of 84% (Table 1).

Different classifiers were tested to establish the best classifier for this dataset. Figure S2A displays the performance of the employed classifiers after applying PLS‐based dimension reduction for LDA, QDA (quadratic discriminant analysis) and LR (logistic regression). LDA demonstrated to be the classifier with the best performance, achieving highest accuracy and sensitivity for ML1 and ML2. LR was also tested, nevertheless, in comparison to LDA, the differences in the performance were minimal. LDA is less prone to overfitting in comparison to QDA and does not require as big sample size to guaranty robustness. While SVM (support vector machine) achieved the highest specificity, it had also the lowest sensitivity. The new proposed hierarchical second level classification model, which employs ML1 and ML2, achieved better classification scores when discriminating tumor and NT bladder tissue as the classical one level classification model approaches, reported recently in fiber‐based Raman bladder diagnosis investigations 34, 55. Figure S2B illustrates the performance comparison for classifying LG and HG by using three different approaches, the level 1 model classification approach using a mean spectra per biopsy did not meet efficient scores to differentiate LG from HG, discriminating with very low accuracy (51%). Due to the heterogeneous nature of some biopsies, applying the classical one‐layer approach with a set of random pixels per biopsy, for example, 80 random pixels, and computing the mean spectra termed mean random pixel (MRP), the outcome improves, but is still too low to be of clinical value. When applying the two‐layer approach, using different sets of MRPs, as employed in this study, the achieved performance provides an improved discrimination between LG and HG tumor with and accuracy of 84%. In order to test the classification performance for tumor and NT tissue differentiation based on the biochemical information of particular bands only, two prominent bands, that is, collagen band (1305 cm^−1^) intensities and lipid band (2850 cm^−1^), were used for the training of a model. The band information alone allowed to achieve a reasonable classification accuracy of 87% (Figure S2C).

### 3.2 Band assignments in Raman spectra of nontumor, low‐ and high‐grade tumor tissue

The mean spectra of the LG and HG samples were calculated with the predicted tumor areas of the biopsies using ML1 and are plotted together with the mean spectra of healthy tissue, in Figure 6. Spectral contributions of collagen and protein bands are resolved at 729, 937, 1003, 1104 and 1265 cm^−1^, where C—C stretching of protein is observed at 729 cm^−1^ and the C—C vibration of collagen backbone is evident at 937 cm^−1^ 56, 57. The presence of phenylalanyl protein at 1003 and 1104 cm^−1^ differs between the mean spectra of LG and HG tumor. Strong presence of amide III of collagen is observed at 1265 cm^−1^, where the band intensity of the NT mean spectrum is higher than the band intensity of both LG and HG tumor mean spectra; those differences in collagen bands were also reported by De Jong et al and Stone et al 37, 58.

Main spectral contributions of lipids are resolved at 1064, 1446, 1656 cm^−1^ and the high wavenumber region at 2850 and 2930 cm^−1^, where an increase in band intensities of the tumor spectra (LG and HG) in comparison with the NT spectrum indicates and increment in lipid content for tumor tissue. In addition, the difference spectra between NT and HG (purple), NT and LG (green), Figure 6, show a higher lipid content in both, LG and HG biopsies, which can be observed in the negative peaks of the bands at 1064, 2850 and 2885 cm^−1^. Significant lipid bands are resolved at the CH_2_ bending mode of lipids and CH_2_ symmetric stretching of protein and lipids evident at 1446, 2850, 2885 and 2930 cm^−1^, respectively 59. The band assignments are summarized in Table 2. Main variations between LG and HG tumor spectra are resolved at protein bands 1176 and 1446 cm^−1^, as well as in the high wavenumber region at 2930 cm^−1^. The spectral features for LG and HG spectra have also been previously reported by Stone et al 58.

**Table 2:** Raman band assignments for spectra in Figure 6 67

Wavenumber (cm^−1^)	Bond assignment	Macromolecules
729	C—C stretching, proline	Collagen 56
869	C—C stretching, choline group	Collagen and lipid 68
937	(C—C) vibration of the collagen backbone	Collagen 56, 57
1003	Phenylalanine, C—C skeletal, phosphate group	Collagen and lipid 57, 68
1064	Skeletal C—C stretch	Lipid 57
1103	Phosphate group and symmetric ring breathing of phenylalanine	Proteins (collagen) and lipid 57, 69
1176	C—H bending tyrosine	Proteins 57
1265	Amide III of collagen, v(CN), d(NH) amide III	Collagen 56, 57, 70
1335	CH_3_CH_2_ wagging	Collagen 18, 57
1446	CH_2_ bending mode of proteins and lipids, CH_2_ deformation	Lipids and proteins 19, 57
1656	C=C lipids, amide I (proteins)	Lipids and proteins 69
2850	υ_s_CH_2_, lipids, fatty acids CH_2_ symmetric	Lipids 57, 59
2885	ν_as_(=CH_2_), ν_s_CH_3_, lipids, fatty acids	Lipids 57, 59
2930	CH_2_ sym. stretching, chain‐end CH_3_ sym. stretching	Protein and lipids 59, 71

### 3.3 Evaluation of biopsy heterogeneity

Tumor heterogeneity can be understood in multiples ways: it can be described in terms of observable features, such as morphology, nanoscale structure 60, cellular arrangement, histology 61, genotypes and protein expression 62. Likewise, it can be studied at different levels, such as molecular, intracellular or bulk levels. A previous study demonstrated that Raman spectroscopic imaging can provide high spatial resolution measurements of the distribution profiles from tissue constituents, such as collagen and glycosaminoglycans in tissue 63 and nucleic acid, protein and lipid in eukaryotic cells 64, 65. Heterogeneity is particularly challenging for classification problems, where heterogeneous data can significantly affect the reliability and performance of the models. Furthermore, for clinical in vivo applications it is important to understand if there is a need to perform Raman imaging to do an accurate characterization of the tumor grading, or if point measurements using, for example, a fiber optic Raman probe suffices. Two approaches were investigated at the bulk level. The first approach uses the classification model's predictions (ML1) to estimate the tumor and NT fraction of the biopsy. The second approach consists in the visual inspection of the distribution of main constituents, collagen, protein and lipids in corresponding Raman images of the biopsy at relevant bands. There is currently no Raman‐based study, which characterizes the heterogeneity of bladder tissue. The presented work considers the intrinsic biomolecular heterogeneity of tumor tissue and attempts to elucidate the molecular fingerprint, which allows to discriminate between the different pathologies.

#### 3.3.1 Heterogeneity of the biopsy based on the prediction of ML1

For classification problems, heterogeneous data can affect the reliability and performance of the models, resulting in reduced sensitivity and specificity. For biopsies, heterogeneity is frequently also related to the fact that the extracted tissue samples not only have the tissue of interest, that is, tumor tissue, but also contain normal tissue located in the proximity of the tumor tissue, or due to the proper orientation of the tissue for measurement. From brightfield images it is impossible to differentiate tumor and NT regions. In consequence, taking spectral information from the entire biopsy can affect the performance of the models, or result in not well reproducible data. It is, therefore, important to find a method to split the heterogeneous data into a set of homogeneous groups of data.

**Figure 7:**
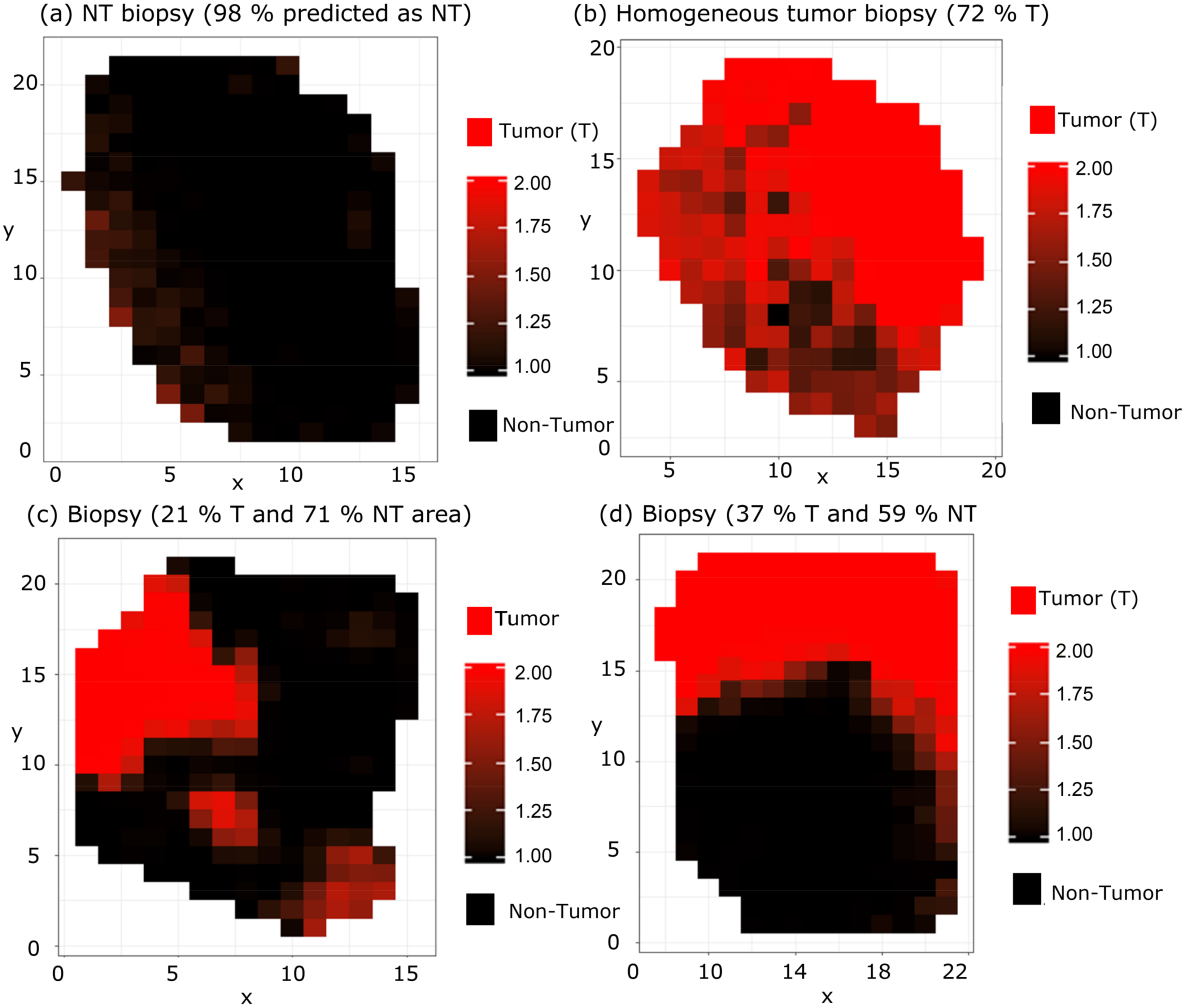
Mean prediction map for tumor and nontumor (NT) regions of test biopsies: (A) NT biopsy 98% of the spectra predicted as NT, (B) homogeneous tumor biopsy with 72% of tumor (T) area, (C) biopsy with 21% of tumor (T) area and 71% of NT area, and (D) test biopsy with 37% of T area and 59% of NT area

There are two key questions, firstly, how the tissue heterogeneity can be bypassed to do a proper classification of tumor grading and secondly how the proposed model generation method can effectively group the data into homogeneous units. To answer these questions, the generated ML1 was employed to predict all the spectra of each tumor biopsy, as it was described in Section 2.3. Each individual spectrum of a biopsy was tested and cross‐validated by the model and a mean of the resulting predictive values was calculated. This mean prediction value was plotted at each location of the hyperspectral image of each biopsy. Four cases are shown in Figure 7, where 1 encodes a purely NT (black) and 2 a purely tumor spectrum (red), respectively. For further analysis we set the boundary condition such that values below 1.5 to NT tissue and values above 1.5 to be tumor tissue. As can be seen in Figure 7 biopsies significantly differ from each other.

For example, the biopsy in Figure 7A,B are very homogeneous, that is, independently of the location of the acquired spectrum ML1 would mostly predict for (A) healthy tissue and (B) tumor tissue. The pathological diagnosis was healthy and HG tumor, respectively. The biopsies mapped in Figure 7C,D, on the other hand, are highly heterogeneous and present multiple NT regions, which if included into the model building would negatively affect the results. From the examples shown here it is clear that many of tumor biopsies actually contain areas that are NT, and if included into the modeling, would substantially influence the performance of the model.

#### 3.3.2 Heterogeneity at the bulk level based on the molecular content in the biopsy

**Figure 8:**
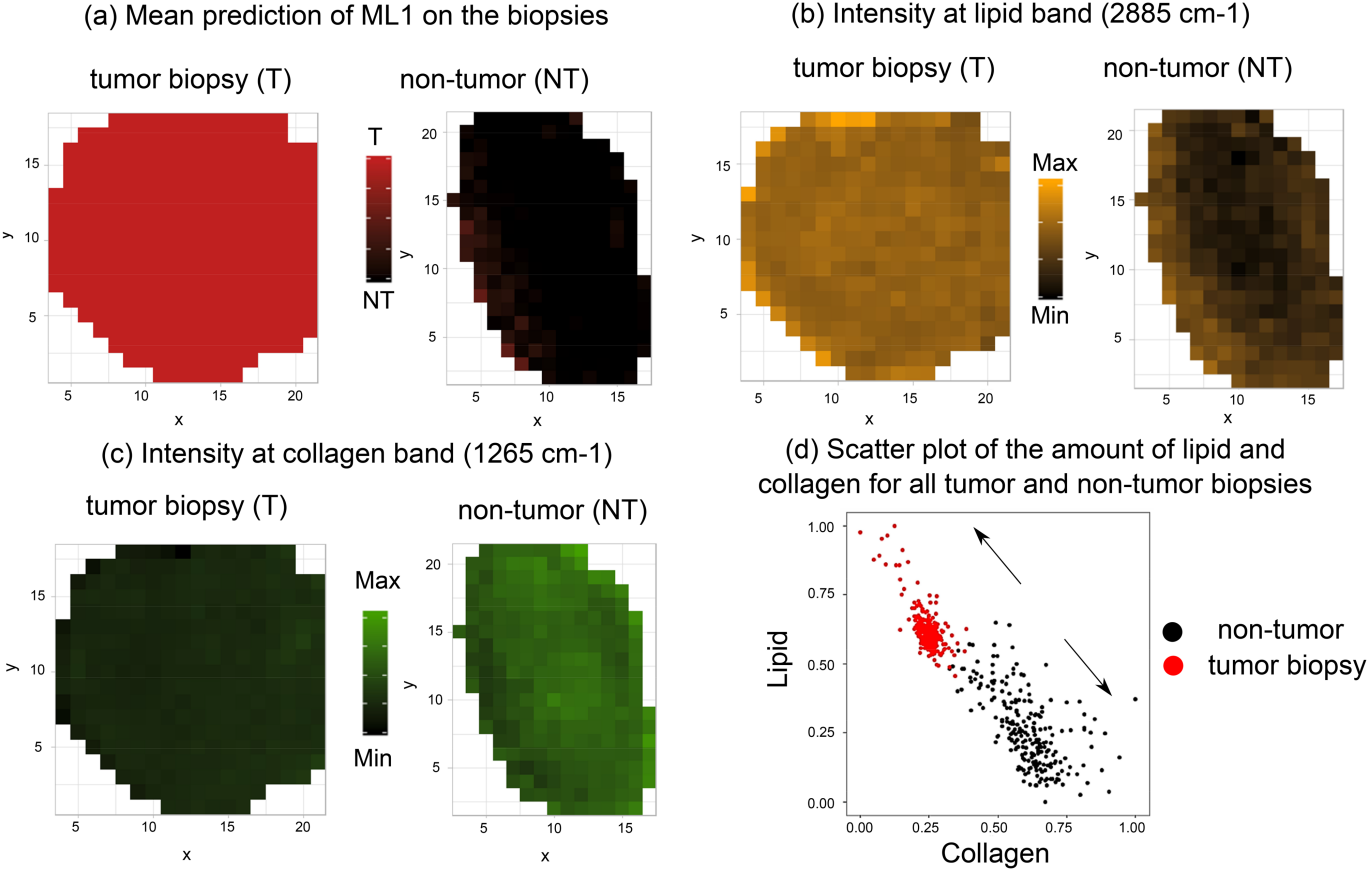
Images of tumor and nontumor based on: (A) mean prediction of the ML1 for homogeneous tumor and nontumor biopsies, where red is predicted as tumor and black as nontumor; Raman intensity image of the same tumor and nontumor tissue at: (B) lipid band (2885 cm^−1^) and (C) collagen band (1265 cm^−1^). The color information represents different chemical constituents, that is, green for collagen and yellow for lipid. The scatter plot (E) of the relative intensity of lipid and collagen for all biopsies shows the relation between the main constituents and the tissue characterization (nontumor and tumor). The intensity of the bands at the mentioned wavenumbers was normalized to min‐max

Raman imaging can be used to learn about the heterogeneity of healthy and tumor bladder tissue at the bulk level by mapping the Raman intensity of specific bands on a biopsy. In Figure 8 the distributions of relevant constituents of the biopsies, that is, collagen (1265 cm^−1^) and lipid (2885 cm^−1^) Raman bands for two selected biopsies diagnosed as high‐grade tumor (left panels) and inflammation (right panels), respectively, are visualized. For comparison, Figure 8A illustrates the mean prediction of ML1 on homogeneous tumor and NT biopsies and Figure 8B,C illustrates the relative Raman intensity of the bands at selected wavenumbers. Based on Figure 8B it can be seen how lipid contributions are more dominant in the tumor biopsy in comparison to the NT biopsy. The presence of collagen is observed in Figure 8C where amide III of collagen exhibits higher intensity in the NT biopsy in contrast to the tumor biopsy. To get a better comprehension of the imaging data a scatter plot of the relative intensity of lipid and collagen from all biopsies was used to depict the relative concentrations of lipid and collagen, Figure 8D. The point clouds were visualized in a 2D scatter plot, which encodes the relative number of points from tumor (red) and NT spectra (black). Consistently, most Raman spectra belonging to NT tissue according to ML1 show higher amounts of collagen in comparison to those from biopsies predicted as tumor. On the other hand, spectra from tumor tissue prove to have higher relative amounts of lipid. Both, Raman images shown in Figure 8A‐C and the scatter plot in Figure 8D provide evidence for significant spectral changes in bladder tissue undergoing tumor development and are consistent with results from biochemical investigations of previous studies 32, 58, 66.

## 4 CONCLUSION

In this study, we demonstrated that Raman spectroscopic imaging employing a hand‐held probe can be used as a valuable tool to characterize bladder tissue at the molecular level. We provide an extensive biochemical characterization of bladder cancer pathology to facilitate real‐time assessment of tumor tissue in future studies. The optical fiber Raman probe imitates the optical performance expected in an in vivo setting, presenting initial operation benchmarks and influential factors to consider for future in vivo investigations. A hierarchical classification was performed, where the first level models (ML1) predict the main differences between tumor and NT tissue and the second level models (ML2) differentiate between HG and LG tumor. The model‐based evaluation has shown that the changes in collagen bands and the increase of the lipid intensity can be associated in differences between tumor and NT tissue, and changes in the protein bands can be used as an indicator to differentiate between LG and HG. The PLS‐LDA models can differentiate tumor from NT with a sensitivity of 92% and a specificity of 93%, while the achieved sensitivity to differentiate LG from HG tumor is 85%. In our selected test group, NT tissue is assigned with an overall accuracy of 92% with confidence levels between 89% and 95%. The LG and HG can be predicted with 84% accuracy in a confidence interval between 78% and 89%. The findings of this study also serve as indication of biopsy heterogeneity, where the prediction of the models, which classify tumor and NT, are used to map the tumor areas on the biopsy. This results in a better performance of the second level models, which use only the tumor areas to train and validate the models to differentiate LG and HG tumors. In addition, the mapping of the intensity at representative lipid, collagen and protein bands of different biopsies served to follow changes of these main constituents. It was demonstrated that a fiber‐based Raman system may complement the well‐established methods, such as cystoscopy, to achieve an immediate bladder tumor diagnosis and thus give the possibility to treat tumor immediately instead of waiting for histopathological diagnosis of a biopsy from the bladder lesion. Ultimately, Raman probe assisted bladder endoscopy can be performed in the outpatient department using small and less traumatizing instruments, resulting in additional health cost savings and significant improvement in patients' prognosis and quality of life. Simultaneously, immediate tumor diagnosis will allow for the instant decision whether the patient can be treated immediately in the outpatient department or needs admittance to the urology ward, as LG noninvasive bladder tumors less than 1.5 cm can be treated in the outpatient department.

## AUTHOR BIOGRAPHIES

Please see Supporting Information online.

## Supporting information


**Table S1**. Primary histopathology by number of patients and biopsies. Miscellaneous refers to nonbladder tumor histopathology.

**Figure S1**. Background corrected Raman spectra (mean ± SD) using different methods to remove fiber background and sample autofluorescence. (A) Extended multiplicative signal correction (EMSC), (B) asymmetric least squares (ALS), (C) polynomial fitting with baseline suppression relative to original spectrum and (D) statistics‐sensitive nonlinear iterative peak‐clipping algorithm (SNIP). The EMSC algorithm achieved the best results removing effectively fiber and fluorescence background.

**Figure S2**. Performance of the classifiers and the classification method.

**Figure S3**. Prediction for the tumor and nontumor location for a typical tumor biopsy, and an indicated number of randomly selected spectra: (A) 1 random spectrum, (B) 15, (C) 30, (D) 40, (E) 60 and (F) 80 random spectra.

**Figure S4**. Mean ± SD of tumor area against the number of spectra randomly selected to build the models for (A) heterogeneous biopsy which area is around 21% tumor and 71% nontumor and (B) homogeneous tumor biopsy with 72% of predicted tumor area. The prediction of the models for the indicated number of spectra is plotted as the ratio of tumor region and total region to the number of spectra used to build the model.
