# Supplementary material for: Bladder tissue characterization using probe‐based Raman spectroscopy: Evaluation of tissue heterogeneity and influence on the model prediction
Source: J Biophotonics. 2019 Dec 2;13(2):e201960025. doi: 10.1002/jbio.201960025 (PMC7065650; doi:10.1002/jbio.201960025)

**Supplementary Materials**

**Bladder tissue characterization using Fiber based Raman spectroscopy: Evaluation of tissue heterogeneity and influence on the model prediction**

Eliana Cordero ^1^, Jan Rüger ^1^, Dominik Marti ^2^, Abdullah S. Mondol ^1^, Thomas Hasselager ^3^, Karin Mogensen ^3^, Gregers G. Hermann ^3^, Jürgen Popp ^1,4^ and Iwan W. Schie ^1,5^

*Corresponding Author: [iwan.schie@leibniz-ipht.de](mailto:iwan.schie@leibniz-ipht.de)

1. Leibniz Institute of Photonic Technology (Leibniz-IPHT), Albert-Einstein-Straße 9, Jena, Germany
2. Department of Photonics Engineering, Technical University of Denmark (DTU), Frederiksborgvej 399, Roskilde, Denmark
3. Gentofte hospital, Department of Urology, Kildegaardsvej 28, Hellerup, Denmark
4. Institute of Physical Chemistry, Friedrich Schiller University Jena, Helmholtzweg 4, 07743, Jena, Germany
5. University of Applied Sciences – Jena, Carl-Zeiss-Promenade 2, 07745 Jena, Germany

**Table S1** Primary histopathology by number of patients and biopsies. Miscellaneous refers to non-bladder tumor histopathology.

|  | **Non-tumor tissue** | **Low Grade** | **High Grade** | **Tumor (non-grading)** | **Miscellaneous** | **Total** |
| --- | --- | --- | --- | --- | --- | --- |
| Overall diagnosis for patients | 6 (21 % ) | 5 (18 % ) | 6 (21 %) | 4 (14%) | 7 (25 % ) | 28 (100%) |
| Biopsy diagnosis | 11* (16 % ) | 13 (19 % ) | 15 (22 % ) | 9 (13 %) | 19** (28 % ) | 67 (100%) |
|  | Number of biopsies (patients) used to calculate the model that differentiate tumor and non-tumor | | | | | |
|  | 11 (6) | 13 (5) | 15 (6) | 9 (4) | 0 | 48 (21) |

*: Benign inflammation was found in 4 biopsies

**: Benign prostatic tissue: 4 biopsies; cancer prostate: 2 biopsies; unknown histopathology: 6 biopsies; different experimental conditions: 7 biopsies of normal tissue.

**Figure S1** Background corrected Raman spectra (mean +/- sd) using different methods to remove fiber background and sample autofluorescence. (a) Extended multiplicative signal correction (EMSC), (b) asymmetric least squares (ALS), (c) Polynomial fitting with baseline suppression relative to original spectrum and (d) statistics-sensitive non-linear iterative peak-clipping algorithm (SNIP). The EMSC algorithm achieved the best results removing effectively fiber and fluorescence background.


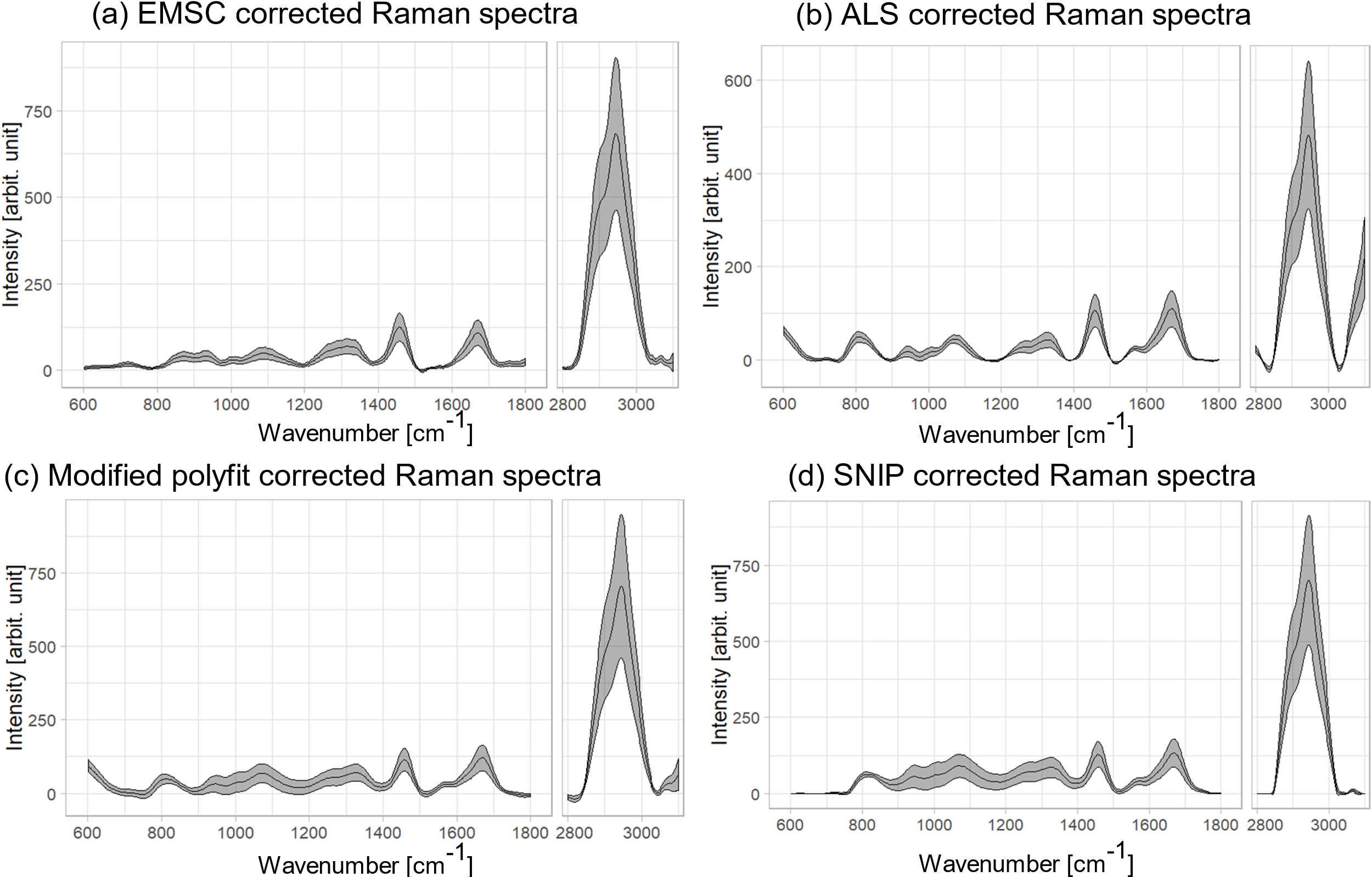


**Figure S2**. Performance of the classifiers and the classification method: (a) Accuracy, sensitivity and specificity for tumor/non-tumor classification of the same mean spectra data set when classifying using logistic regression (LR), partial least squares/ linear discriminant analysis (PLS/LDA), Quadratic discriminant analysis (QDA) and support vector machine (SVM). The variations in the performance are minimal, nevertheless, PLS/LDA achieved higher accuracy and sensitivity. (b) Classification method to discriminate low and high grade tumor, the 1 level model classification (1LMC) using a mean spectrum per biopsy in light gray is the simplest solution achieving very low classification performance, the 1 level model classification using the mean spectra of 80 random pixels (1LMC-MRP) in gray and the 2 level model classification using the mean spectra of 1-80 random pixels (2LMC-MRP) in dark gray presents the best performance. (c) LDA ML1 classification of tumor/non-tumor tissue based on the biochemical content of the biopsies, the intensities of collagen band (1305 cm^-1^) and lipid band (2850 cm^-1^)are used to create the model by selecting 40 random spectra to account for the spatial variance of lipid and collagen.

**
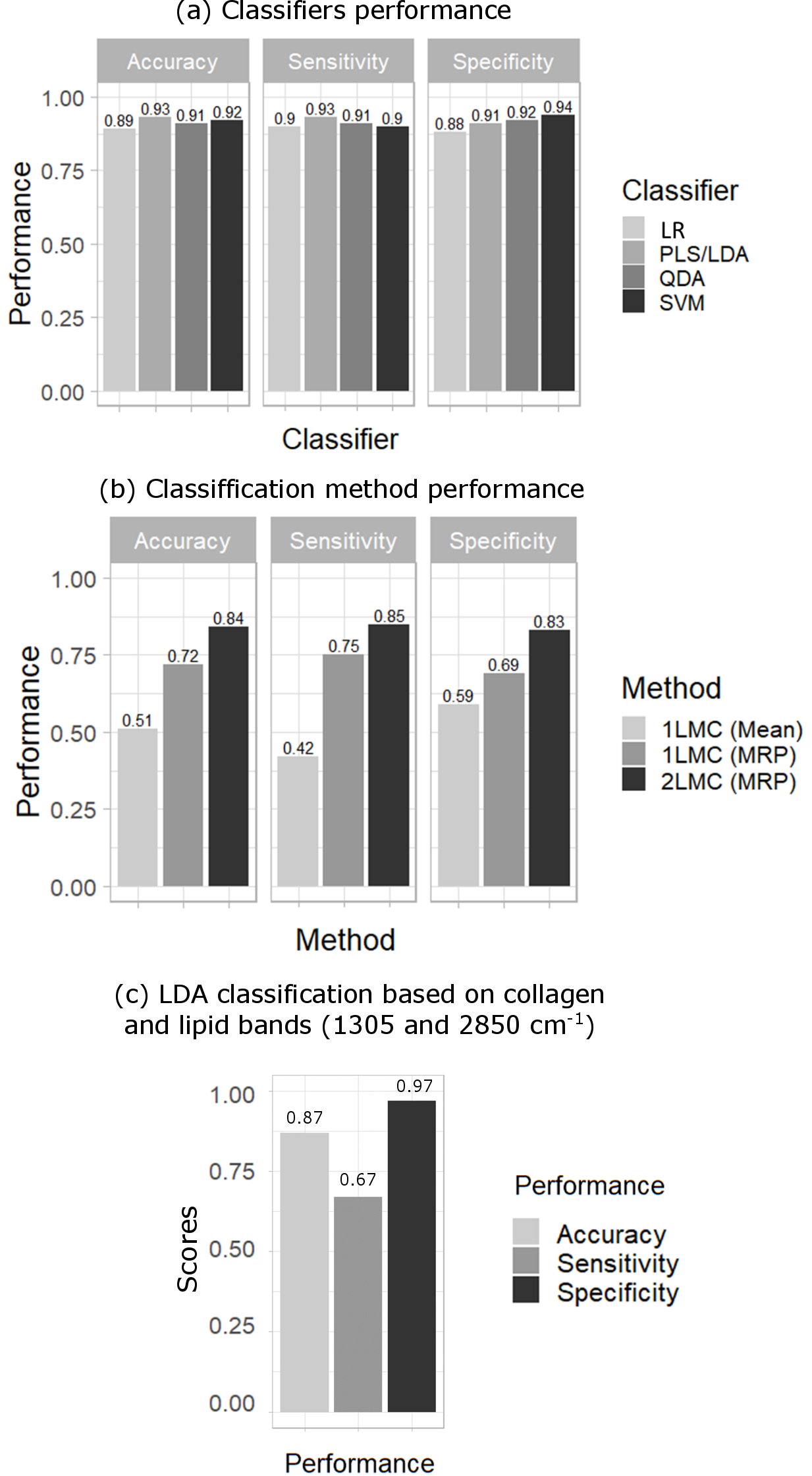
**

**Figure S3**. Prediction for the tumor and non-tumor location for a typical tumor biopsy, and an indicated number of randomly selected spectra: (a) 1 random spectrum, (b) 15, (c) 30, (d) 40, (e) 60 and (f) 80 random spectra.


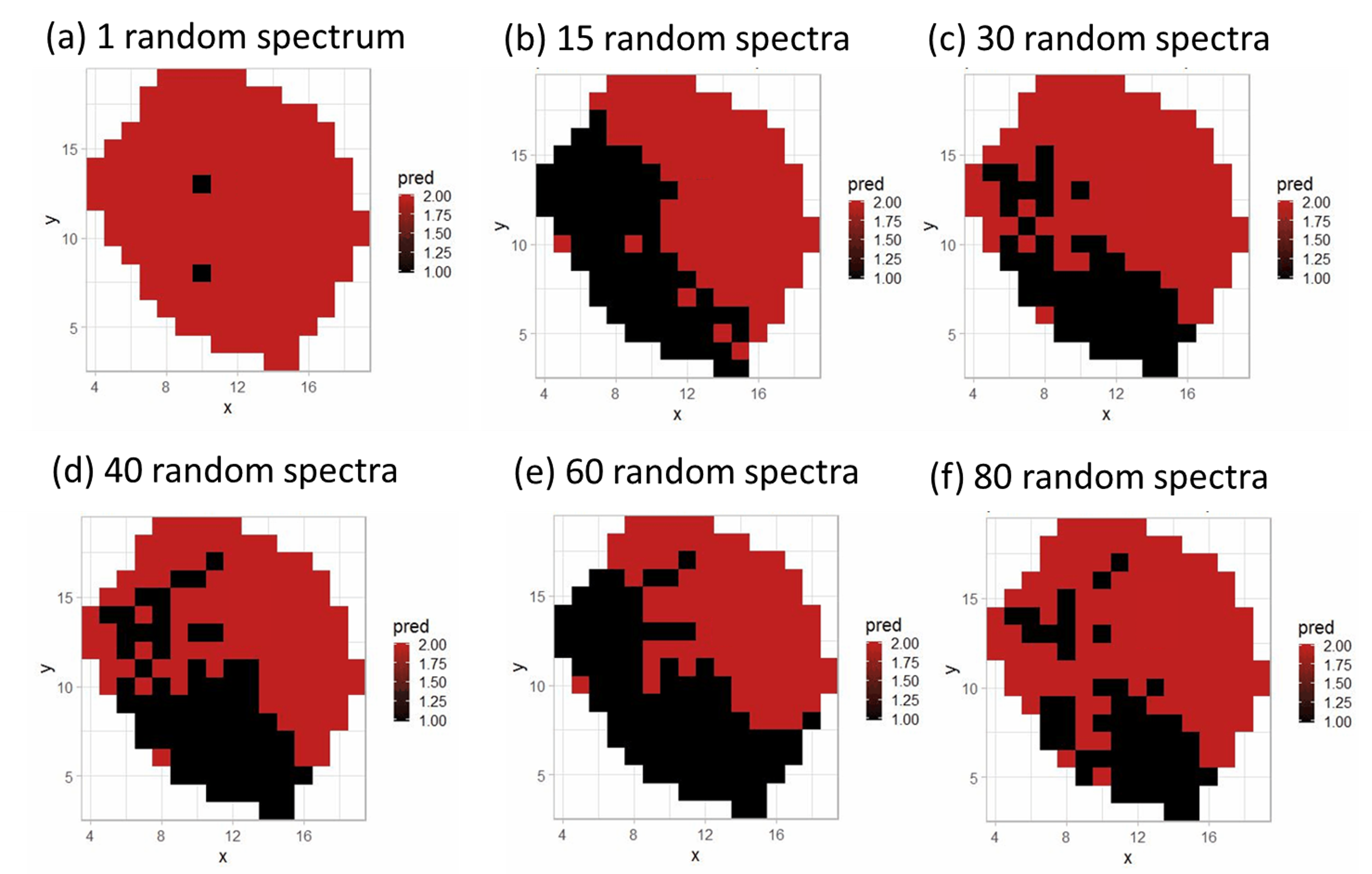


**Figure S4** Mean-sd of tumor area against the number of spectra randomly selected to build the models for (a) heterogeneous biopsy which area is around 21 % tumor and 71 % non-tumor and (b) homogeneous tumor biopsy with 72 % of predicted tumor area. The prediction of the models for the indicated number of spectra is plotted as the ratio of tumor region and total region to the number of spectra used to build the model.


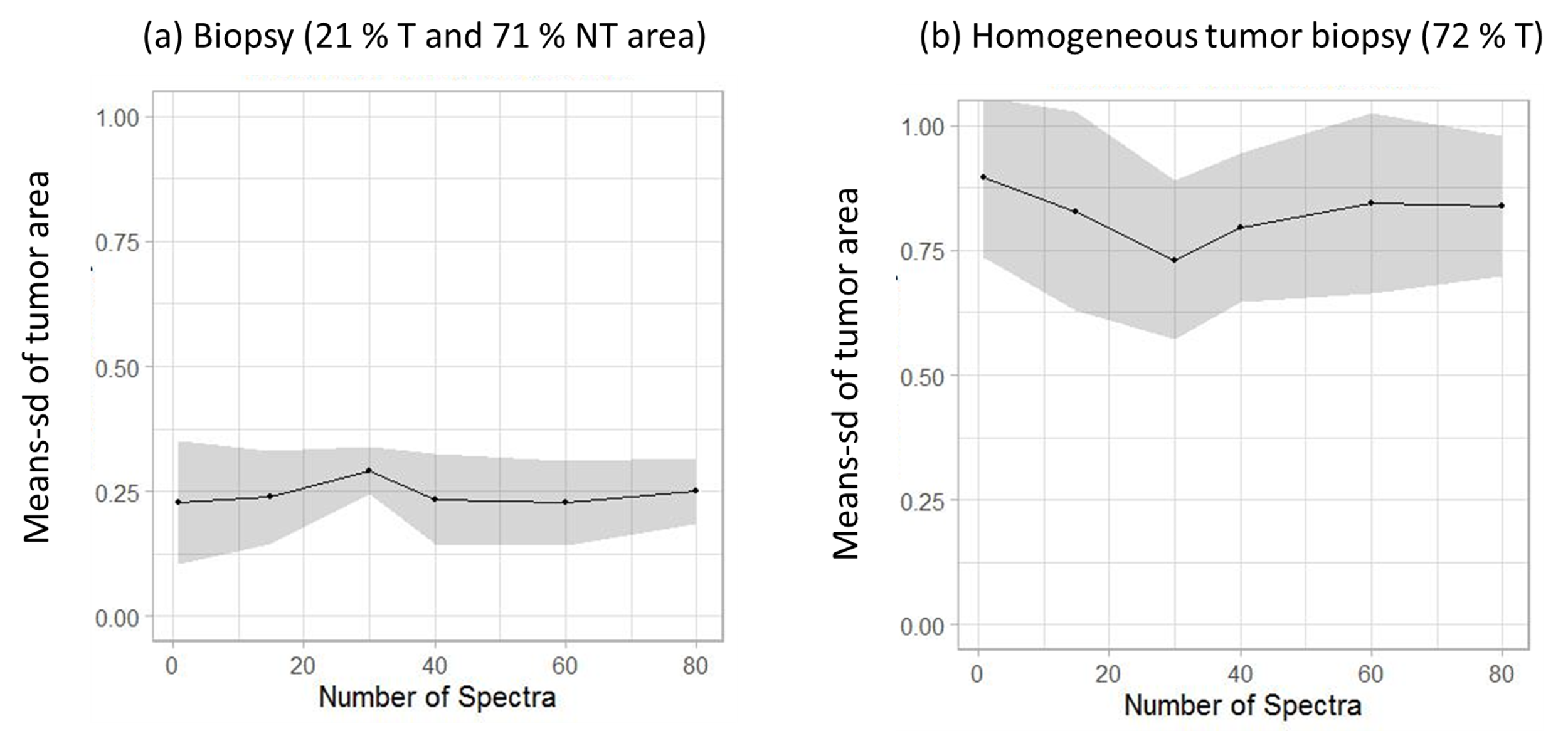

Supplement: Supplementary file 1 — Table S1. Primary histopathology by number of patients and biopsies. Miscellaneous refers to nonbladder tumor histopathology. [file JBIO-13-e201960025-s002.docx]
